# Supplementary material for: Inflammatory Signals Induce AT2 Cell-Derived Damage-Associated Transient Progenitors that Mediate Alveolar Regeneration
Source: Cell Stem Cell. 2020 Sep 3;27(3):366–382.e7. doi: 10.1016/j.stem.2020.06.020 (PMC7487779; doi:10.1016/j.stem.2020.06.020)
Supplement: Document S1. Figures S1–S7 [file mmc1.pdf]

Cell Stem Cell, Volume *27*

Supplemental Information

Inflammatory Signals Induce AT2 Cell-Derived Damage-Associated Transient Progenitors that Mediate Alveolar Regeneration

Jinwook Choi, Jong-Eun Park, Georgia Tsagkogeorga, Motoko Yanagita, Bon-Kyoung Koo, Namshik Han, and Joo-Hyeon Lee

Supplemental Information

Inflammatory Signals induce AT2 Cell-Derived Damage-Associated Transient Progenitors that Mediate Alveolar Regeneration

Jinwook Choi, Jong-Eun Park, Georgia Tsagkogeorga, Motoko Yanagita, Bon-Kyoung Koo, Namshik Han, and Joo-Hyeon Lee

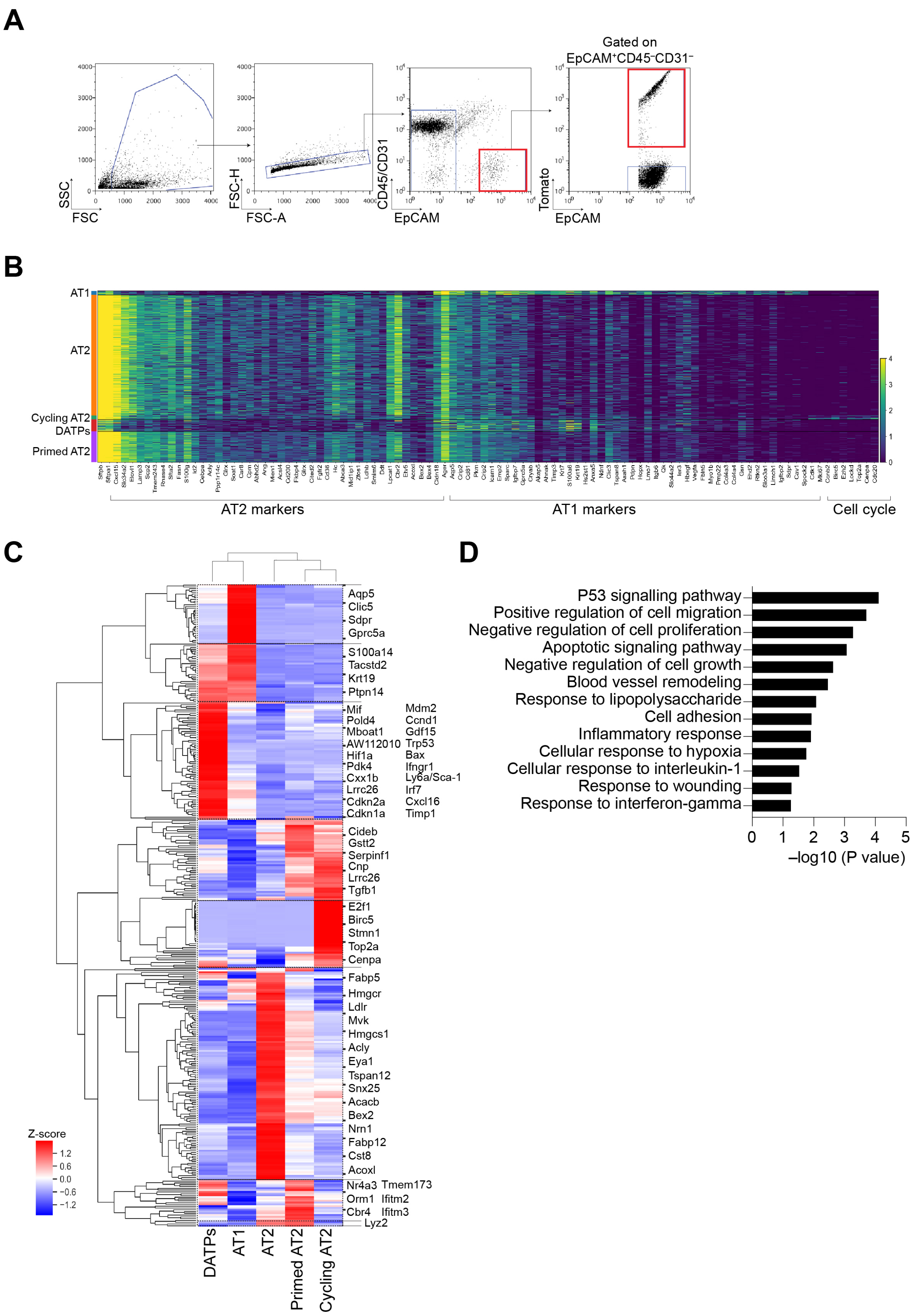

Figure S1, *related to Fig. 1*. Single-cell profiling of *SPC* lineage-labeled cells during injury repair.

(A) Sorting strategy for *SPC* lineage-labeled cells by flow cytometry after bleomycin injury.

(B) Gene expression of AT2 markers, AT1 markers, or cell cycle markers across single cells from distinctive subsets revealed by single-cell RNA sequencing (scRNA-seq) analysis during injury repair.

(C) Heap map showing relative expression of marker genes in distinctive subsets revealed by scRNA-seq analysis.

(D) GO analysis of enriched genes in DATPs.

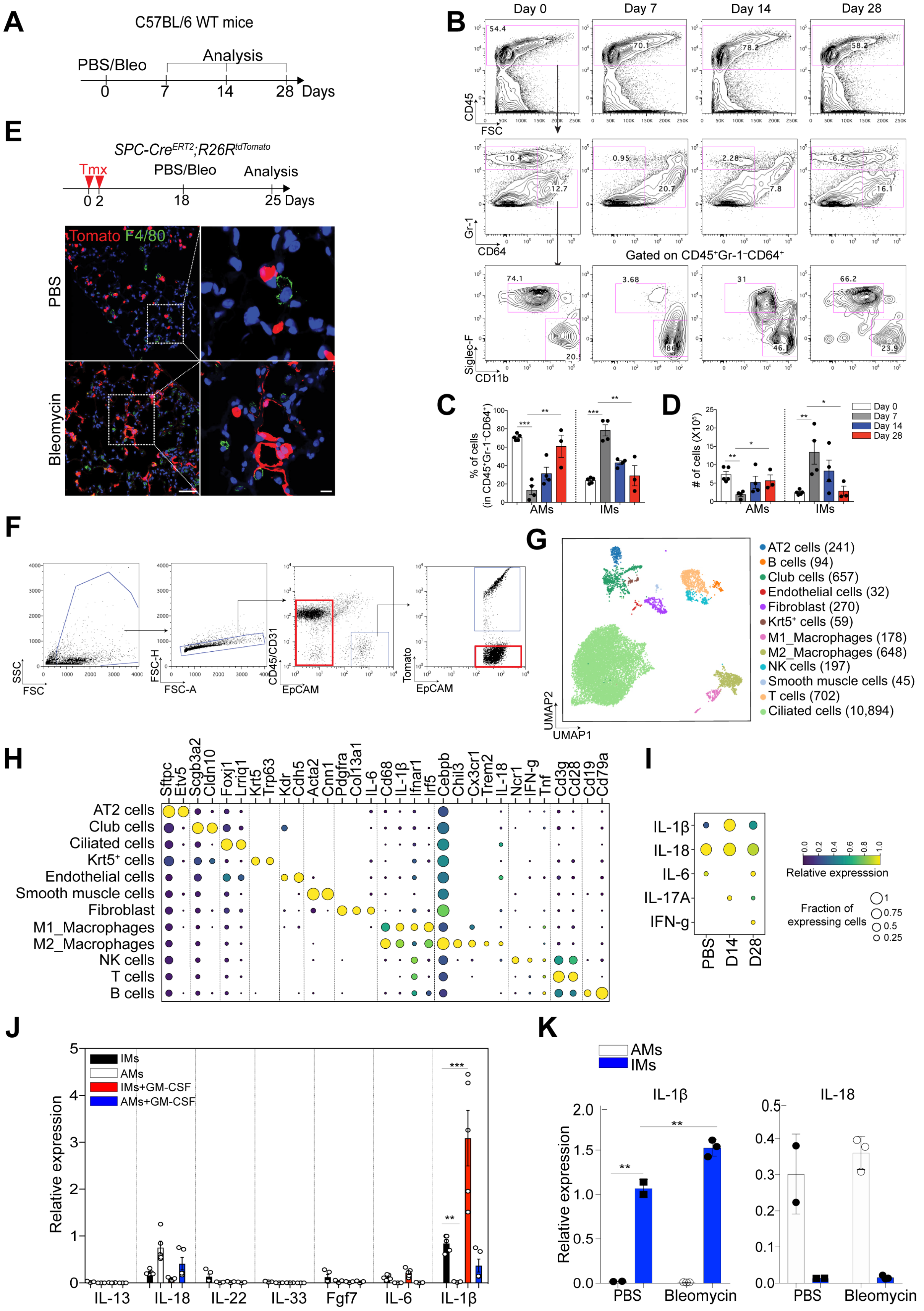

Figure S2, ***related to Fig. 2, A-E*****. Dynamics of macrophages during alveolar regeneration after bleomycin injury.**

(A) Schematic of experimental design for analysis of immune cells at indicated time points after bleomycin injury.

(B) Flow cytometry analysis of alveolar (Siglec-F^{+}CD11b^{low}) and interstitial (Siglec-F^{-}CD11b^{high}) macrophages at indicated time points post injury. Cells gated on CD45^{+}CD64^{+}Gr-1^{-} were analyzed further for expression of Siglec-F and CD11b. Numbers adjacent to the outlined area indicate the percentage of populations.

(C, D) Frequencies (**C**) and absolute cell numbers (**D**) of alveolar (AMs) or interstitial (IMs) macrophages at indicated time points. Each individual dot represents one experiment and date are presented as mean ± SEM. *p<0.05, **p<0.01, and ***p<0.001.

(E) Experimental design (top) of *SPC* lineage-tracing and immunofluorescent (IF, bottom) images of tissue samples after bleomycin treatment. IF images show the increased numbers of F4/80^{+} macrophages at day 7 post injury. A high magnification images (right) show the interaction between macrophages and *SPC* lineage-labeled cells. Data are the representative of two independent experiments. Scale bar, 50 μm (left) and 10 μm (right). Tomato (red), F4/80 (green), and DAPI (blue).

(F) Sorting strategy for *SPC* unlabeled single cells pooling of EpCAM^{+}Tomato^{-} and EpCAM^{-} population by flow cytometry after bleomycin injury.

(G) Clusters of unlabeled cells (14,017) after bleomycin injury from 10xGenomics 3' scRNA-seq analysis visualized by UMAP, assigned by specific colors. Number of cells in the individual cluster is depicted in the figure.

(H) Gene expression of key markers in each distinctive cluster. *IL-1β* is specifically expressed in macrophages.

(I) Gene expression of *IL-1β*, *IL-18*, *IL-6*, *IL-17A*, and *IFN-g* at indicated time points after bleomycin injury. Of note, the expression of *IL-1β* is dramatically increased at day 14 post injury and returns back to the homeostatic level at day 28 post injury.

(J) qPCR analysis of specific cytokine expression in alveolar (AMs) or interstitial (IMs) macrophages in response to activation by GM-CSF. Isolated subsets of macrophages were cultured in the presence or absence of GM-CSF for 24hrs *in vitro*. Each individual dot represents one experiment and date are presented as mean ± SEM.

(K) qPCR analysis for *IL-18* and *IL-1β* in alveolar (AMs, white bar) or interstitial (IMs, blue bar) macrophages isolated at day 7 after PBS or bleomycin treatment. Each individual dot represents one experiment from one mouse and date are presented as mean ± SEM. **p<0.01, ***p<0.001.

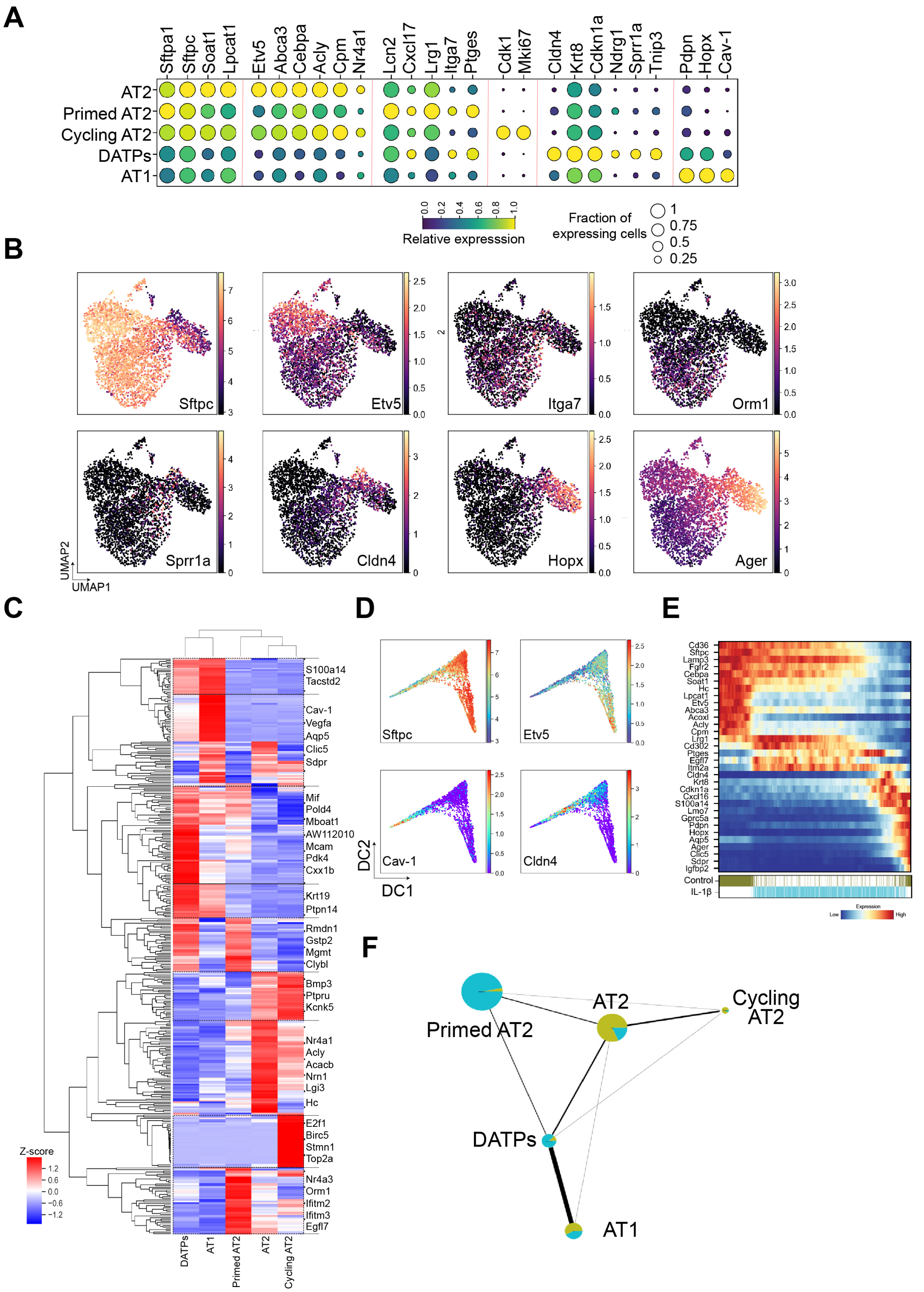

Figure S3, *related to Fig. 2, F-H*. Alveolar organoids challenged by IL-1β recapitulate the behavior of regenerating AT2 cells during injury repair.

(A) Gene expression of key markers in each distinctive cluster.

(B) UMAP visualization of the log-transformed (log_{10}(TPM+1)), normalized expression of selected marker genes in distinctive clusters.

(C) Heap map showing relative expression of selected genes that are specifically expressed in distinctive clusters revealed by scRNA-seq analysis.

(D) Diffusion map according to diffusion pseudotime order colored by expression (log_{10}(TPM+1)) of specific genes.

(E) Gene expression profiles of control and IL-1β-treated organoids ordered according to pseudotime trajectory. Lower color bars indicate annotation by samples.

(F) Network topology among clusters from single cell data revealed by Partition-based graph abstraction (PAGA). Colors indicate the proportion of each cluster by time point. Each node in the PAGA graph represents a cluster and the weight of the lines represents the statistical measure of connectivity between clusters.

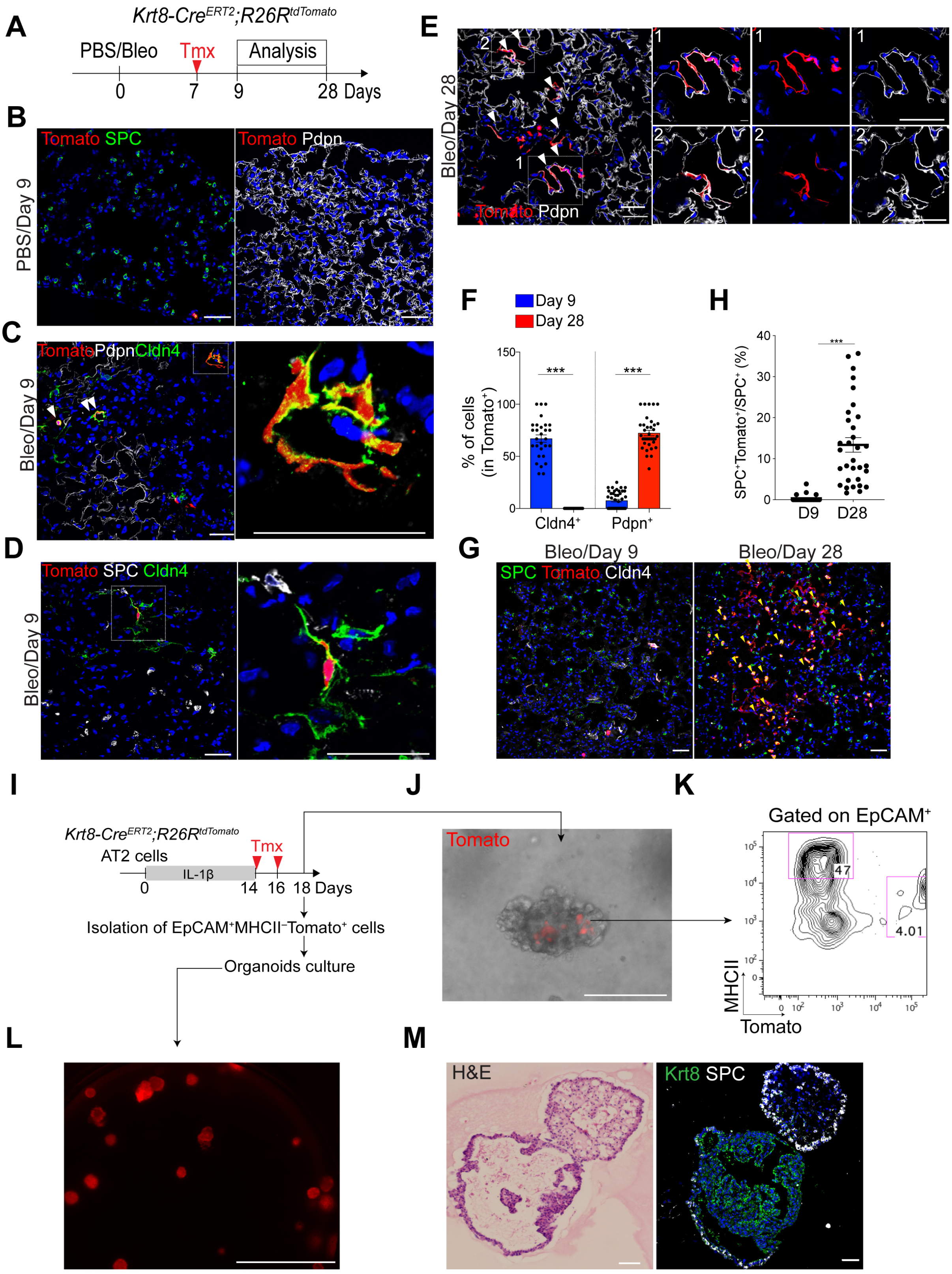

Figure S4, *related to Fig. 3*. Lineage tracing analysis of *Krt8*+ cells reveals that DATPs are capable of producing AT1 cells and reverting to AT2 cells during alveolar regeneration.

(A) Experimental design for *Krt8* lineage-tracing analysis using *Krt8-Cre*^{ERT2}*;R26R*^{tdTomato} reporter mice after bleomycin injury. Specific time points for tamoxifen injection and analysis are indicated.

(B) Representative IF images show that none of AT2 (left) and AT1 (right) cells are lineage-labeled by *Krt8* expression in uninjured lung (PBS control): Tomato (red), SPC (green, left), Pdpn (white, right), and DAPI (blue). Scale bar, 50 μm.

(C, D) Representative IF images show that *Krt8* lineage-labeled cells express Cldn4 at day 9 post injury. None of AT1 (**C**) and AT2 (**D**) cells are lineage-labeled by *Krt8* expression at this time point: Tomato (red), Pdpn (white), Cldn4 (green), and DAPI (blue). Arrowhead points to *Krt8* lineage-labeled DATPs. White boxed insets are shown on the right. Scale bar, 50 μm.

(E) Representative IF images show that *Krt8* lineage-labeled cells generate new AT1 cells at day 28 after injury: Tomato (red), Pdpn (white), and DAPI (blue). Arrowhead points to lineage-labeled Pdpn$^+$ cells. Insets (left) show high-power view (1, right top; 2, right bottom). Scale bar, 50 μm.

(F) Statistical quantification of Cldn4$^+$Tomato$^+$ or Pdpn$^+$Tomato$^+$ cells at indicated time points after injury. Each individual dot represents one section and data are presented as mean ± SEM with two independent experiments (n=5). ***p<0.001.

(G) Representative IF images show that *Krt8* lineage-labeled cells generate AT2 cells at day 28 post injury. Notably, there are few AT2 cells that are marked by *Krt8* expression at day 9 post injury: Tomato (for *Krt8* lineage, red), SPC (green), Cldn4 (white), and DAPI (blue). Arrowhead points to lineage-labeled AT2 cells. Scale bars, 50 μm.

(H) Quantification of *Krt8* lineage-labeled SPC$^+$ AT2 cells. Each individual dot represents one section and data are presented as mean ± SEM with three independent experiments (n=4). ***p<0.001.

(I) Scheme of experimental design for organoid culture assays. AT2 cells were isolated by surface markers CD31$^-$CD45$^-$EpCAM$^+$MHCII$^+$ from *Krt8-Cre*^{ERT2}*;R26R*^{tdTomato} mice and cultured as organoids with IL-1β for 14 days. 4-OH tamoxifen was added at day14 and day16 in culture to label *Krt8*-expressing cells. At day 18, organoids were further analyzed for a microscopy (I), flow cytometry (J), and organoid formation (K and L).

(J) Representative merged fluorescent and brightfield image of organoids in (H). Treatment of 4-OH tamoxifen allows to mark *Krt8*$^+$ (Tomato$^+$) cells. Scale bar, 200 μm. Notably, Tomato signals were detected only in inner parts of organoids.

(K) Flow cytometry analysis of AT2 ($EpCAM^{+}MHCII^{+}Tomato^{-}$) and DATPs ($EpCAM^{+}MHCII^{-}Tomato^{+}$) from dissociated organoids in (I). Numbers adjacent to the outlined area indicate the percentage of populations. Of note, $Tomato^{+}$ cells are not AT2 cells. **(L, M)** Representative fluorescent image (**L**), and H&E staining (**M**, left) and IF image (**M**, right) of organoids derived from dissociated *Krt8*$^{+}$Tomato^{+} cells in (I and J). Scale bar, 1,000 μm (**L**) and 50 μm (**M**).

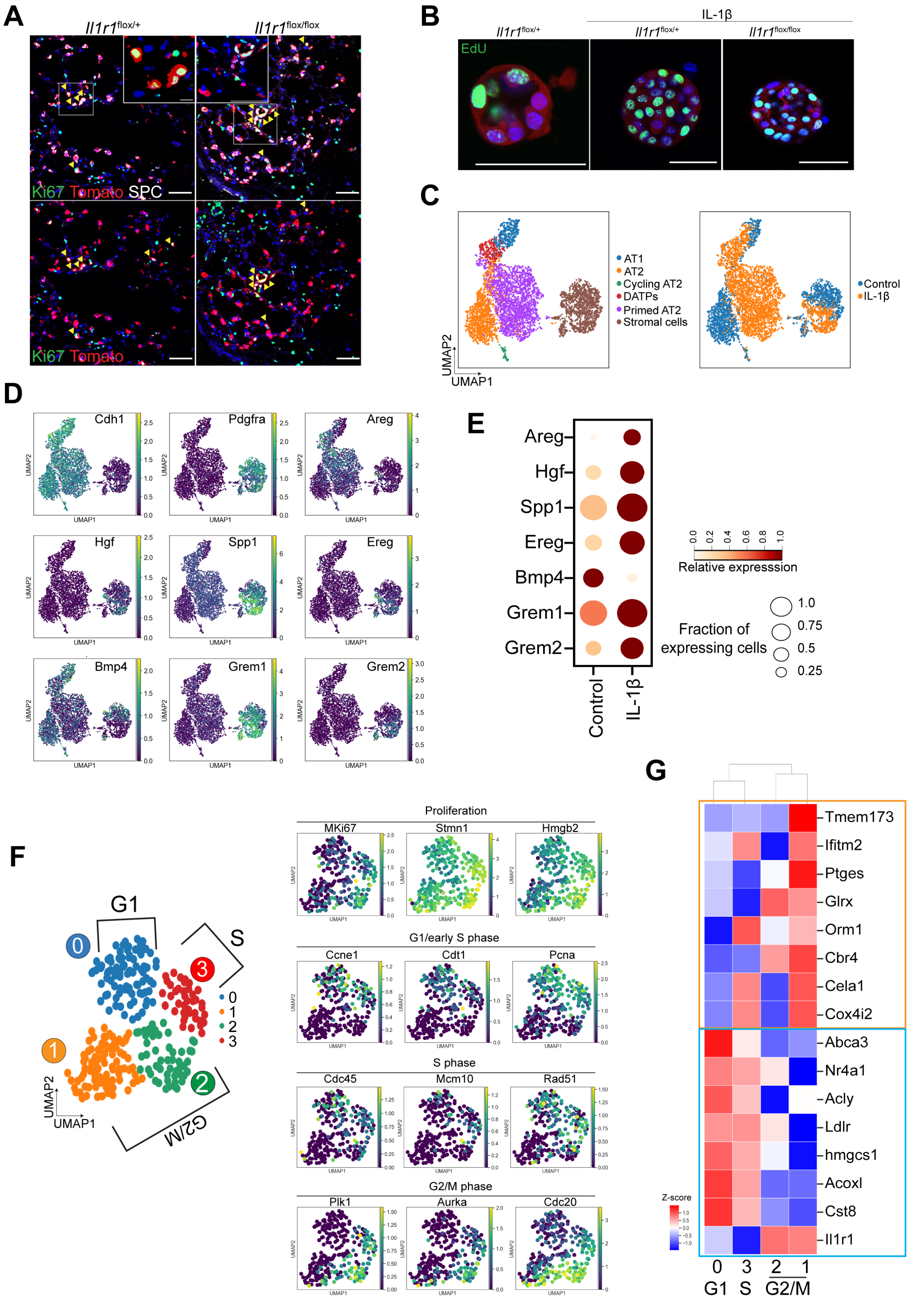

Figure S5, *related to Fig. 4, A-E*. IL-1β signaling primes AT2 cells during cell cycle transition.

(A) Representative IF images showing Ki67$^+$ lineage-labeled AT2 cells in the lung of mice treated with PBS or bleomycin at day 7 post injury: Tomato (for *SPC* lineage, red), Ki67 (green), SPC (white), and DAPI (blue). Arrowheads, Ki67$^+$ AT2 cells. Insets show high-power view. Scale bars, 50 μm. No discernible differences in number of Ki67$^+$ AT2 cells were observed in the lung of indicated genotyped mice.

(B) Representative IF images showing proliferating cells in AT2 organoids derived from the lungs of indicated genotyped mice. Organoids were pulsed with BrdU for 4hrs at day 4 in cultures. Notably, IL-1β treatment enhances proliferation in organoids regardless of *Il1r1* expression in AT2 cells.

(C) UMAP visualization of cell clusters from scRNA-seq analysis of epithelial cells and stromal cells from control or IL-1β-treated organoids. Cells were isolated at day 21 in organoid culture. Colors indicate distinct cell types (left) and samples (right).

(D) UMAP visualization of the log-transformed ($\log_{10}$(TPM+1)), normalized expression of cell type marker genes (e.g. *Cdh1* for epithelial cells and *Pdgfra* for stromal cells/fibroblast) and growth factors in each distinctive cluster.

(E) Gene expression of growth factors that may enhance proliferation of AT2 cells in control or IL-1β-treated stromal cells.

(F) Clusters of Cycling AT2 population (cAT2) shown in Fig. 1B visualized by UMAP, assigned by specific colors. Based on the expression of cell cycle genes, four clusters were classified into two cell cycle phases; G1 (cluster 0), S phase (cluster 3) and G2/M phase (cluster 2 and 1). UMAP visualization of the log-transformed ($\log_{10}$(TPM+1)), normalized expression of marker genes for cell proliferation and cell cycle (G1/early S phase; S phase; G2/M phase).

(G) Heap map showing the *Il1r1* expression and acquisition of Primed AT2 cell (pAT2) signatures during cell cycle transition. Acquisition of transcriptional signatures of pAT2 cells by downregulating of naïve AT2 cell markers including *Abca3* (blue box) and inducing expression of genes related with inflammatory response including *Ptges* (orange box) during cell cycle transition from S to G2/M phase.

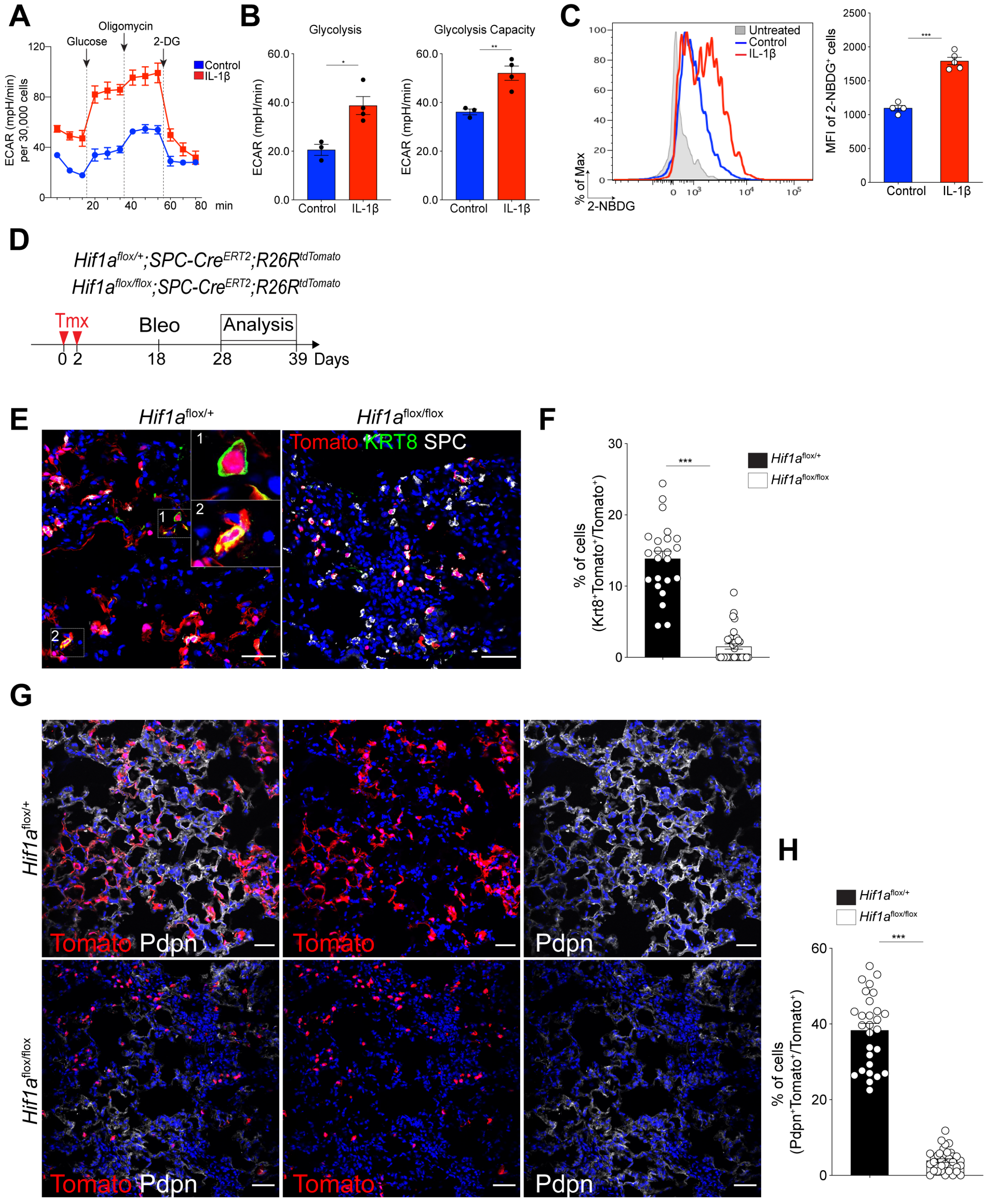

Figure S6, *related to Fig.4, F-I.* Deletion of *Hif1a* on AT2 cells impairs DATPs generation and AT1 cell regeneration.

(A) Real-time ECAR (Extracellular Acidification Rate) of organoids treated with PBS (control) and IL-1β was measured by XF-96 analyzer. Vertical lines with arrow indicate addition of glucose (glycolysis substrate, 10mM), oligomycin (ATP synthase inhibitor, 1uM), and 2-Deoxy Glucose (2-DG, glycolysis inhibitor, 50mM). X axis indicates measurement times.

ECAR was normalized to 30,000 cells. data are presented as mean ± SE (n=3 for control; n=4 for IL-1β).

(B) Representative graphs output from XF96 anlyzer showing the glycolysis (left) and glycolytic capacity (right). *p<0.05, and **p<0.01.

(C) Effects of IL-1β on glucose uptake. 2-NBDG incorporation from organoids treated with PBS control (blue line) or IL-1β (red line) was determined by flow cytometry (left). Non-treated cells were used as a negative control for 2-NBDG treatment (grey-filled peak). Representative histograms showing MFI (mean fluorescence of intensity) of 2-NBDG (right). Each individual dot represents one individual experiment and data are presented as mean ± SEM (n=4 for control; n=5 for IL-1β). ***p<0.001.

(D) Experimental design for lineage tracing. Date for analysis is as indicated.

(E) Representative IF images showing *SPC* lineage-labeled DATPs at day 14 post injury in the lung of indicated genotyped mice: Tomato (for *SPC* lineage, red), Krt8 (green), SPC (white), and DAPI (blue). Insets (left) show high-power view (right top). Scale bars, 50μm.

(F) Quantification of *SPC* lineage-labeled DATPs in (**B**). Each individual dot represents one section and data are presented as mean ± SEM with three independent experiments. Notably, there is a significant decrease in number of lineage-labeled DATPs in the absence of *Hif1a* in AT2 cells.

(G) Representative IF images showing AT1 cell differentiation from *SPC* lineage-labeled cells at day 28 post injury in the lung of indicated genotyped mice: Tomato (for *SPC* lineage, red), Pdpn (white), and DAPI (blue). Scale bars, 50 μm.

(H) Quantification of lineage-labeled $Pdpn^{+}$ AT1 cells in (**D**). Each individual dot represents one section and data are presented as mean ± SEM (n=3 for $Hif1a^{flox/+}$; n=4 for $Hif1a^{flox/flox}$). Notably, there is a significant decrease in the number of lineage-labeled AT1 cells in the absence of *Hif1a* in AT2 cells.

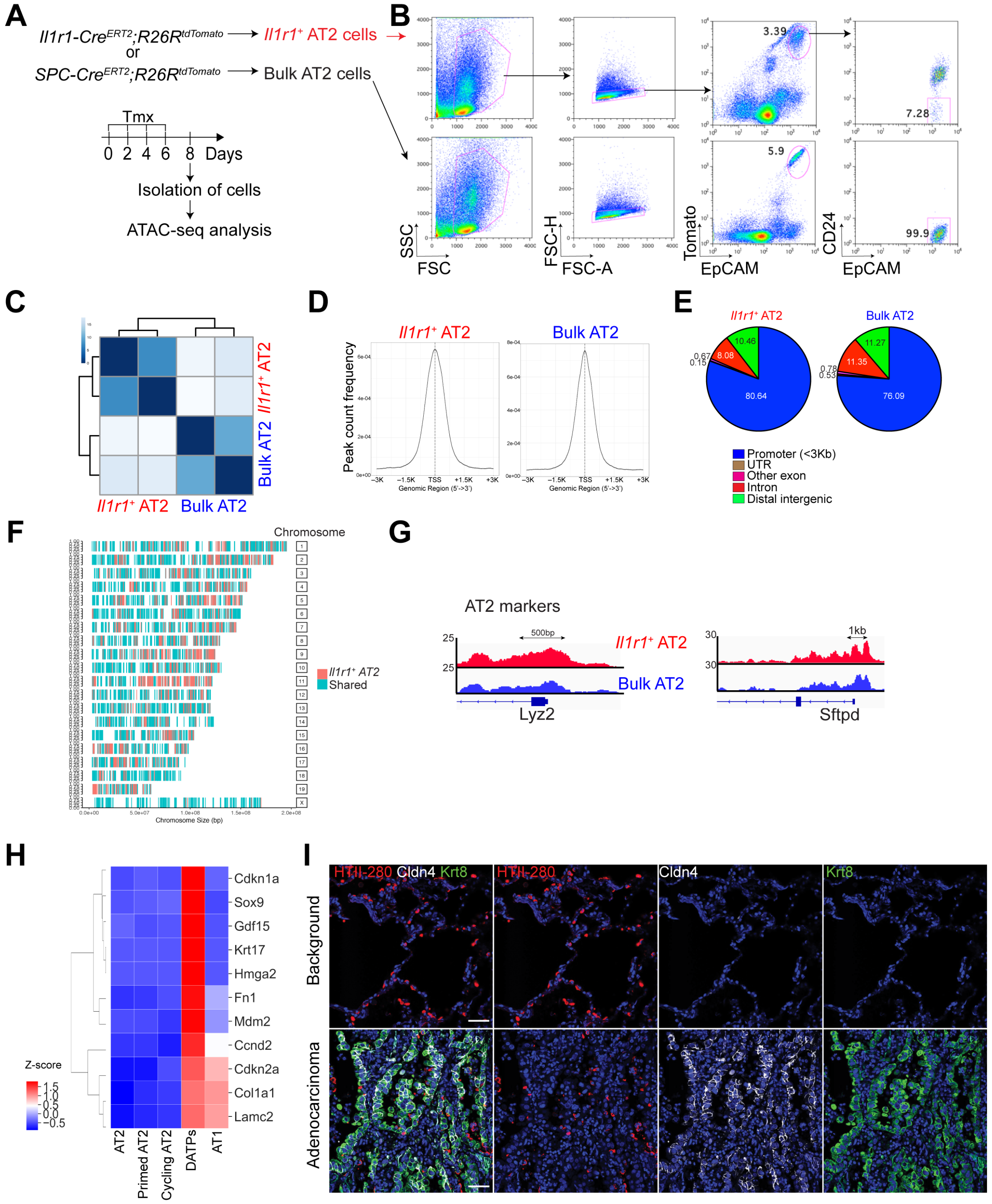

Figure S7, *related to Fig. 6 and 7*. ATAC-seq analysis showing distinct differences in open chromatin structure in *Il1r1*$^{+}$AT2 cells versus bulk AT2 cells and aberrant accumulation of DAPT-like population in the lung from adenocarcinoma patients.

(A, B) Experiment design (**A**) and sorting strategy by flow cytometry (**B**) for isolating *Il1r1*$^{+}$AT2 or bulk AT2 cells from *Il1r1-Cre*^{ERT2}*;R26R*^{tdTomato} or *SPC-Cre*^{ERT2}*;R26R*^{tdTomato} mice, respectively.

(C) Heat map of poisson distances between samples on the original count matrix.

(D) Density plots depicting enrichment of ATAC-seq signals at TSSs ± 3 kb.

(E) Distribution of ATAC-seq peaks within defined genomic regions of predicted mRNAs. UTR, untranslated regions.

(F) Genome-wide profiling of ATAC-seq peaks in *Il1r1*$^+$AT2 and bulk AT2 cells.

(G) Snapshots of peaks enriched in shared genes Lyz2 and Sftpd. Arrows denote direction of transcription.

(H) Heat map of the transcriptional profiles of genes that are highly expressed in Krt17$^+$ basal-like cells in IPF patients in the subset of clusters.

(I) Representative IF images of KRT8$^+$CLDN4$^+$ cells in the lung from adenocarcinoma patients (n=3). HTII-280 (red), CLDN4 (white), KRT8 (green) and DAPI (blue). Background region (top) in the lung tissue of the same patient was used for control. Scale bar, 50 μm.
